# Transcriptomic and proteomic analyses of genetic factors influencing adductor muscle coloration in QN Orange scallops

**DOI:** 10.1186/s12864-019-5717-y

**Published:** 2019-05-09

**Authors:** Junlin Song, Chunde Wang

**Affiliations:** 1 Qingdao Agricultural University, Qingdao, 266109 China; 2 Yantai Institute of Coastal Zone Research, Chinese Academy of Sciences, Yantai, 264003 China

**Keywords:** Transcriptome, Proteome, Adductor muscle color, QN Orange scallop

## Abstract

**Background:**

Color polymorphism, a high-valued trait, is frequently observed in molluscan shellfish. The QN Orange scallop, a new scallop strain successively selected from the interspecific hybrids of the bay scallop (*Argopecten irradians irradians*) and the Peruvian scallop (*Argopecten purpuratus*), is distinguished from other scallops by its orange adductor muscles. In this study, to reveal the mechanisms of the formation of adductor muscle coloration in the QN Orange scallops, we compared the proteome and transcriptome of orange adductor muscles of the QN Orange and those of white adductor muscles of the Bohai Red scallop, another strain selected from the interspecific hybrids of the bay scallop and the Peruvian scallop.

**Results:**

Transcriptomic analysis revealed 416 differentially expressed genes (DEGs) between white and orange adductor muscles, among which 216 were upregulated and 200 were downregulated. Seventy-four differentially expressed proteins (DEPs), including 36 upregulated and 38 downregulated proteins, were identified through label-free proteomics. Among the identified DEGs and DEPs, genes related to carotenoids biosynthesis including *apolipophorin*, and Cytochrome P450 and those related to melanin biosynthesis including *tyrosinase* and Ras-related protein *Rab-11A* were found to express at higher levels in orange adductor muscles. The high expression levels of *VPS* (vacuolar protein sorting) and *TIF* (translation initiation factor) in orange adductor muscle tissues indicated that carotenoid accumulation may be affected by proteins outside of the carotenoid pathway.

**Conclusions:**

Our results implied that the coloration of orange adductor muscles in the QN Orange scallops may be controlled by genes modulating accumulation of carotenoids and melanins. This study may provide valuable information for understanding the mechanisms and pathways underlying adductor muscle coloration in molluscan shellfish.

**Electronic supplementary material:**

The online version of this article (10.1186/s12864-019-5717-y) contains supplementary material, which is available to authorized users.

## Background

Color polymorphism, a high-valued trait that appeals to consumers, is common in molluscan shellfish [1], and more variations in color are observed in shell color than in softbody color. Previous studies on coloration in shellfish showed that color polymorphism is inheritable and may be tightly regulated by a set of genes [2–4]. To increase the product value, numerous varieties have been selected on either shell color or softbody color in molluscan shellfish such as the Pacific abalone (*Haliotis discus hannai*), the Pacific oyster (*Crassostrea gigas*), and the Japanese pearl oyster (*Pinctada fucata martensii*) [5–7].

In molluscan shellfish, variations in color are often caused by the presence of different pigments such as pyrroles, melanins, bile, and porphyrins [8, 9]. Carotenoids are the most seen porphyrins in animals. Existing evidence suggests that orange coloration in some mollusks is the result of carotenoid accumulation in these animals [8], although presence of melanins also leads to orange coloration in other animals [10, 11]. Carotenoids in most animals are taken from diets as they are not able to synthesize carotenoids endogenously. Carotenoid accumulation in the adductor muscles of the yesso scallop (*Patinopecten yessoensis*), the bay scallop (*Argopecten irradians irradians*), and the noble scallop (*Chlamys nobilis*) have been reported [12–14]. These carotenoids included pectenolone and pectenoxanthin as seen in the yesso scallop [14, 15]. In the yesso scallop, one genomic region and two SNPs have been found to be associated with coloration as revealed by genome-wide association studies [15]. In the bay scallops, 126 single-nucleotide polymorphisms (SNPs) related to carotenoid accumulation have been detected by Genotyping-by-Sequencing analysis [12]. Despite these efforts, the mechanism underlying carotenoid accumulation in molluscs remains poorly understood and we are still not sure whether other pigments or genetic factors are also involved in adductor muscle coloration.

**Fig. 1:**
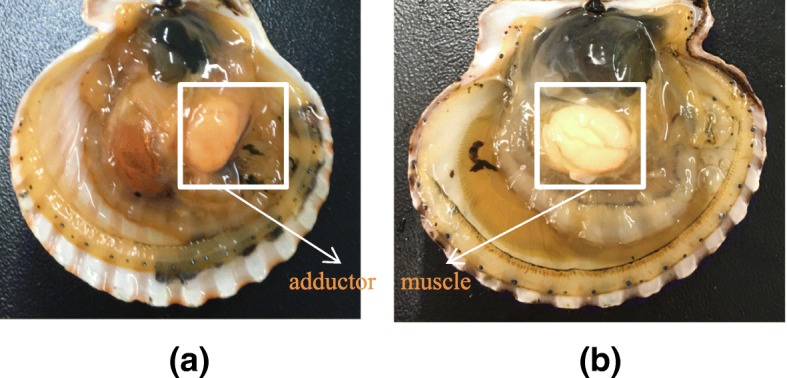
The adductor muscles of QN Orange scallops and Bohai Red scallops. **a** QN Orange scallops with orange adductor muscles; **b** Bohai Red scallops with adductor white muscles

In our previous studies, two scallop strains, the QN Orange and Bohai Red (Fig. 1), have been selected from the interspecific hybrids of the bay scallop and the Peruvian scallop (*Argopecten purpuratus*). Unlike Bohai Red whose adductor muscles are white, the QN Orange strain was intentionally selected for its orange adductor muscle. It has been found that high contents of pectenolone and pectenoxanthin are present in the QN Orange strain (unpublished). As both strains were selected from the same origin cohort and share the same genetic background, they may thus provide excellent opportunity for studies on adductor muscle coloration in molluscs. In this study, we aimed to identify the potential genes that may be involved in scallop adductor muscle coloration based on transcriptomic and label-free proteomic analyses.

## Results

### Transcriptomic analysis

Illumina sequencing yielded 321,347,360 raw reads (GEO accession number: GSE122451). A total of 157,546,736 clean reads were obtained for white adductor muscles with Q20 values of 96.74–98.33% and 163,800,624 clean reads were obtained for orange adductor muscles with Q20 values of 97.99–98.08%. The total size of the clean data of each sample exceeded 7.0 Gb (Additional file 1: Table S1). Moreover, 91.78–92.63% of the clean reads from white adductor muscles and 92.24–92.75% of the clean reads from orange adductor muscles were aligned to the bay scallop genome (Additional file 2: Table S2).

**Fig. 2:**
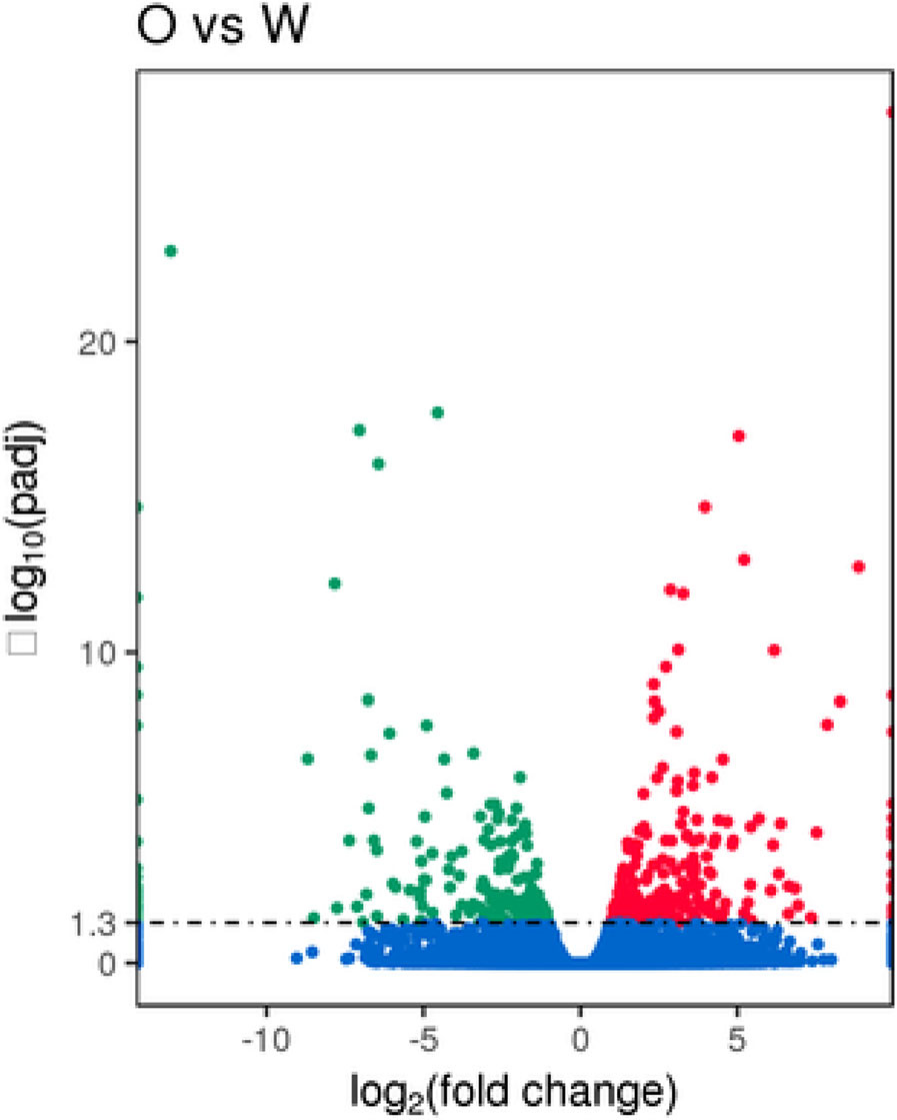
Volcano plots for differentially expressed genes (DEGs) between orange and white adductor muscle samples. Points of the plots represent genes that are significantly differentially expressed. Green points represent up regulated genes of orange adductor muscles, while red circles represent down regulated genes of white adductor muscles. Abscissa represents multiple genes expressed in different samples change; ordinate represents the statistically significant differences in gene expression quantity change. Padj represents the adjusted *p* values. O vs W stands for orange adductor muscle samples versus white adductor muscle samples

**Fig. 3:**
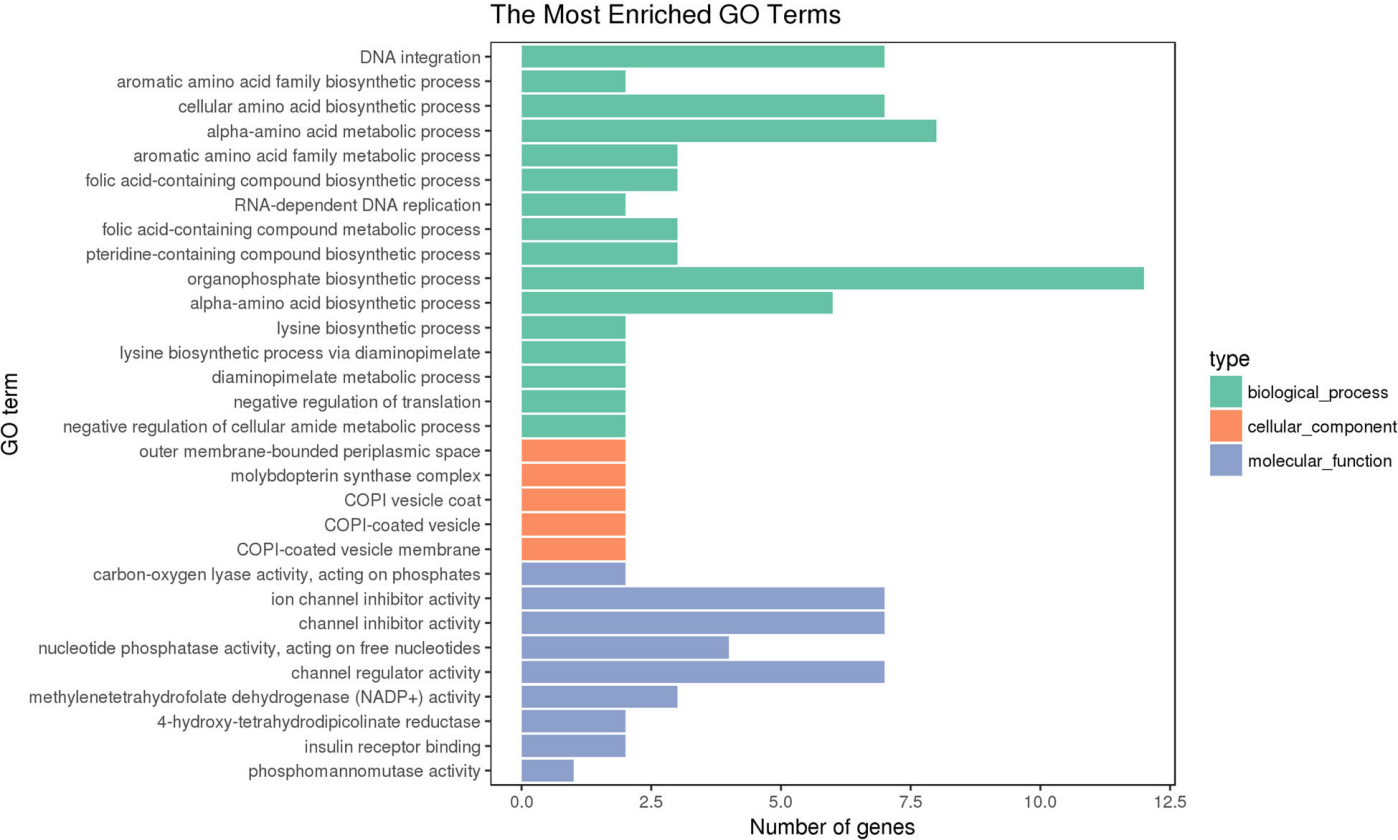
GO distributions of differentially expressed genes (DEGs) from orange and white adductor muscles transcriptomes. GO enrichment of genes and proteins based on biological process (green bar), cellular component (orange bar) and molecular function (blue bar). Abscissa shows the number of genes distributing into different GO terms

**Fig. 4:**
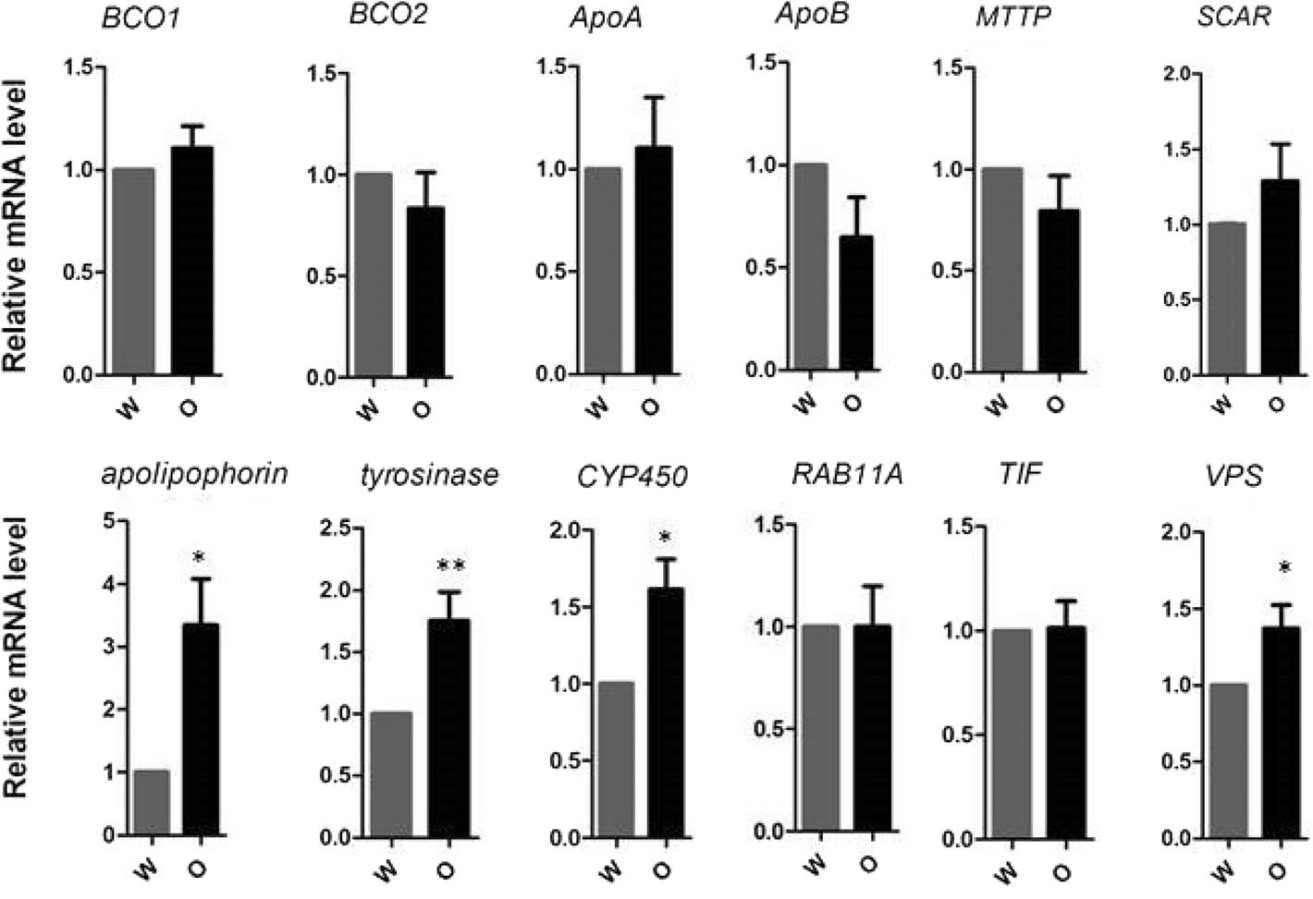
The mRNA expression levels of 12 genes examined by RT-PCR. Expression levels were normalized to β-actin and presented as relative expression to controls (mean ± SD). *W* stands for white adductor muscle samples (gray bar). *O* stands for orange adductor muscle samples (black bar). *P < 0.05; ** *P* < 0.01. *BCO1*, β-carotene15, 15′-monooxygenase; *BCO2*, β-carotene-9, 10′-oxygenase; *ApoA*, apolipoprotein A; *ApoB,* apolipoprotein B; *MTTP*, microsomal triglyceride transfer protein; *SCAR*, scavenger receptors; *CYP450*, cytochrome P450; *RAB11A*, ras-related protein Rab-11A; *TIF*, translation initiation factor; *VPS*, vacuolar protein sorting

A total of 416 DEGs, including 216 upregulated and 200 downregulated genes, were identified between the white adductor muscles of the Bohai Red scallops and the orange adductor muscle of the QN Orange scallops (Fig. 2). GO functional analyses revealed that most of the DEGs were assigned to DNA integration, cellular amino acid biosynthetic process, channel inhibitor activity, channel inhibitor activity, ion channel inhibitor activity, and organophosphate biosynthetic process, as shown in Fig. 3. KEGG pathway analyses of DEGs showed that the most enriched pathways are carbon metabolism, fructose and mannose metabolism, neuroactive ligand–receptor interaction, amino acid biosynthesis, and glycolysis. Two carotenoid-related genes, *apolipophorin* and cytochrome P450 (*CYP450*), and one melanin-related gene, *tyrosinase*, were found to be differentially expressed in the white and orange adductor muscles, which were subsequently verified by RT PCR analysis (Fig. 4).

Notably, transcriptome data showed that genes known to be related to carotenoid deposition, including β-carotene15, 15′-monooxygenase (*BCO1*), β-carotene-9, 10′-oxygenase (*BCO2*), microsomal triglyceride transfer protein (*MTTP*), apolipoprotein A (*ApoA*), apolipoprotein B (*ApoB*), and scavenger receptors (*SCAR*) were not differentially expressed in two kinds of adductor muscles. These results were also verified with RT PCR (Fig. 4).

### Proteomic analysis

**Fig. 5:**
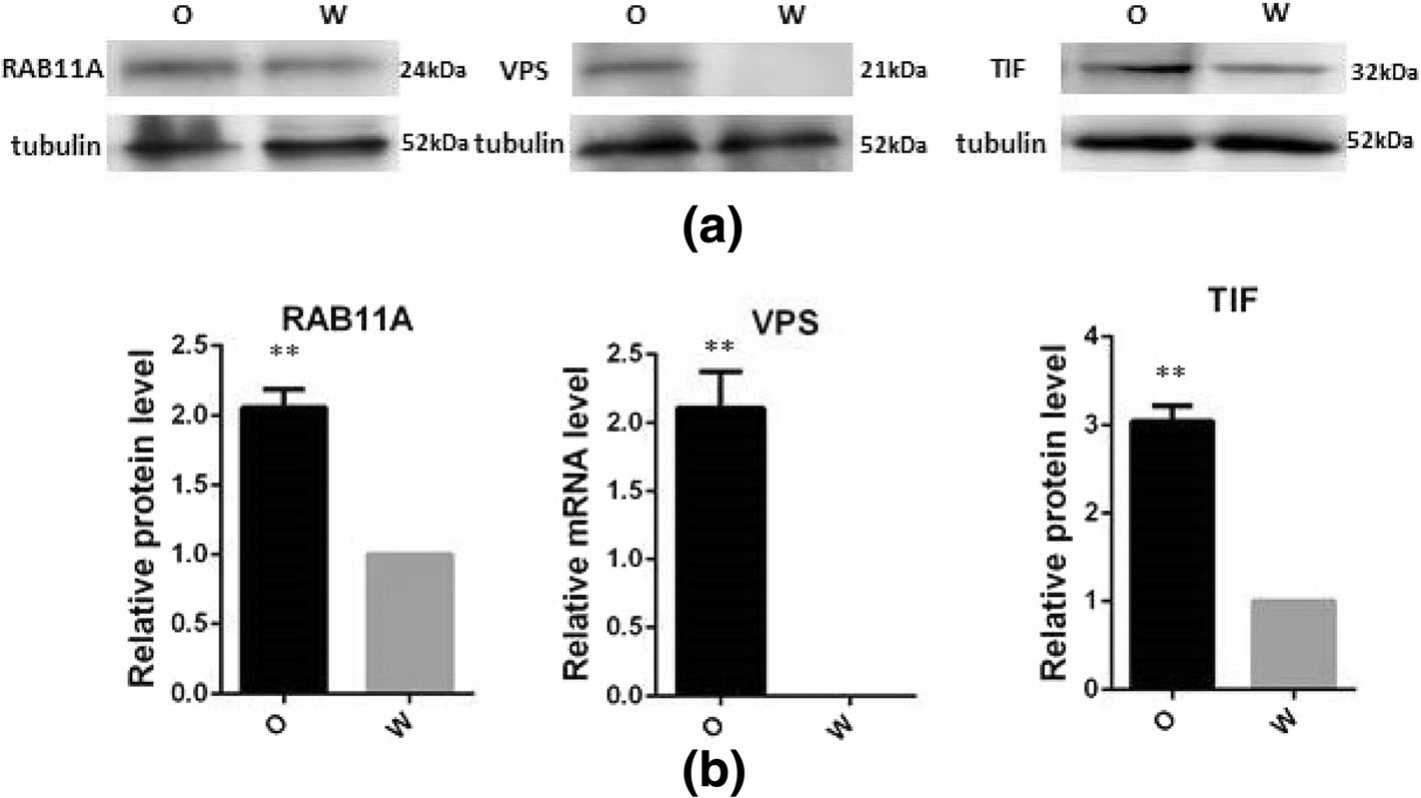
Western blot analysis of *RAB11A, VPS* and *TIF*. **a** SDS-PAGE of *RAB11A*, *VPS* and *TIF*; left of the band was the protein name; right of the band was molecular weight of protein. **b** Protein expression levels of *RAB11A*, *VPS* and *TIF* examined by band intensity. Tubulin was selected as the internal control. W stands for white adductor muscle samples. O stands for orange adductor muscle samples. *P < 0.05; ** *P* < 0.01. *RAB11A*, ras-related protein Rab-11A; *TIF*, translation initiation factor; *VPS*, vacuolar protein sorting

Label-free proteomics yielded 8498 unique peptides and 1154 protein groups (ProteomeXchange identifier PXD011708). A total of 74 proteins were identified to be differentially expressed with 26 upregulated and 24 downregulated in orange adductor muscles, and 10 only present in orange and 14 only in adductor muscles (Additional file 3: Table S3). All DEPs were enriched in 9 GO terms including sulfur amino acid metabolic process, methionine metabolic process, carbohydrate metabolic process, nucleoside metabolic process, hydrolase activity, acting on glycosyl bonds, aspartate family amino acid metabolic process, metal ion binding, and cation binding, as shown in Table 1. Moreover, the most enriched KEGG pathways were other glycan degradation, Kaposi’s sarcoma-associated herpes virus infection, lysosome, VEGF signaling pathway, and cellular senescence. Three proteins related to carotenoid or melanin accumulation were identified to be differentially expressed in the white and orange adductor muscles, namely Ras-related protein Rab-11A *(RAB11A*), translation initiation factor (*TIF*) and vacuolar protein sorting (*VPS*). These results were confirmed by Western-blotting using antibodies against *RAB11A, TIF,* and *VPS* (Fig. 5).

**Table 1:** Enriched GO categories of differentially expressed protein

GO ID	Term	Protein Count	*P* value
GO:0000096	sulfur amino acid metabolic process	3	0.013
GO:0006555	methionine metabolic process	3	0.013
GO:0005975	carbohydrate metabolic process	17	0.023
GO:0009116	nucleoside metabolic process	4	0.024
GO:1901657	glycosyl compound metabolic process	4	0.025
GO:0016798	hydrolase activity, acting on glycosyl bonds	4	0.025
GO:0009066	aspartate family amino acid metabolic process	4	0.025
GO:0046872	metal ion binding	68	0.034
GO:0043169	cation binding	70	0.041

### Transcriptome and proteome association analysis

**Fig. 6:**
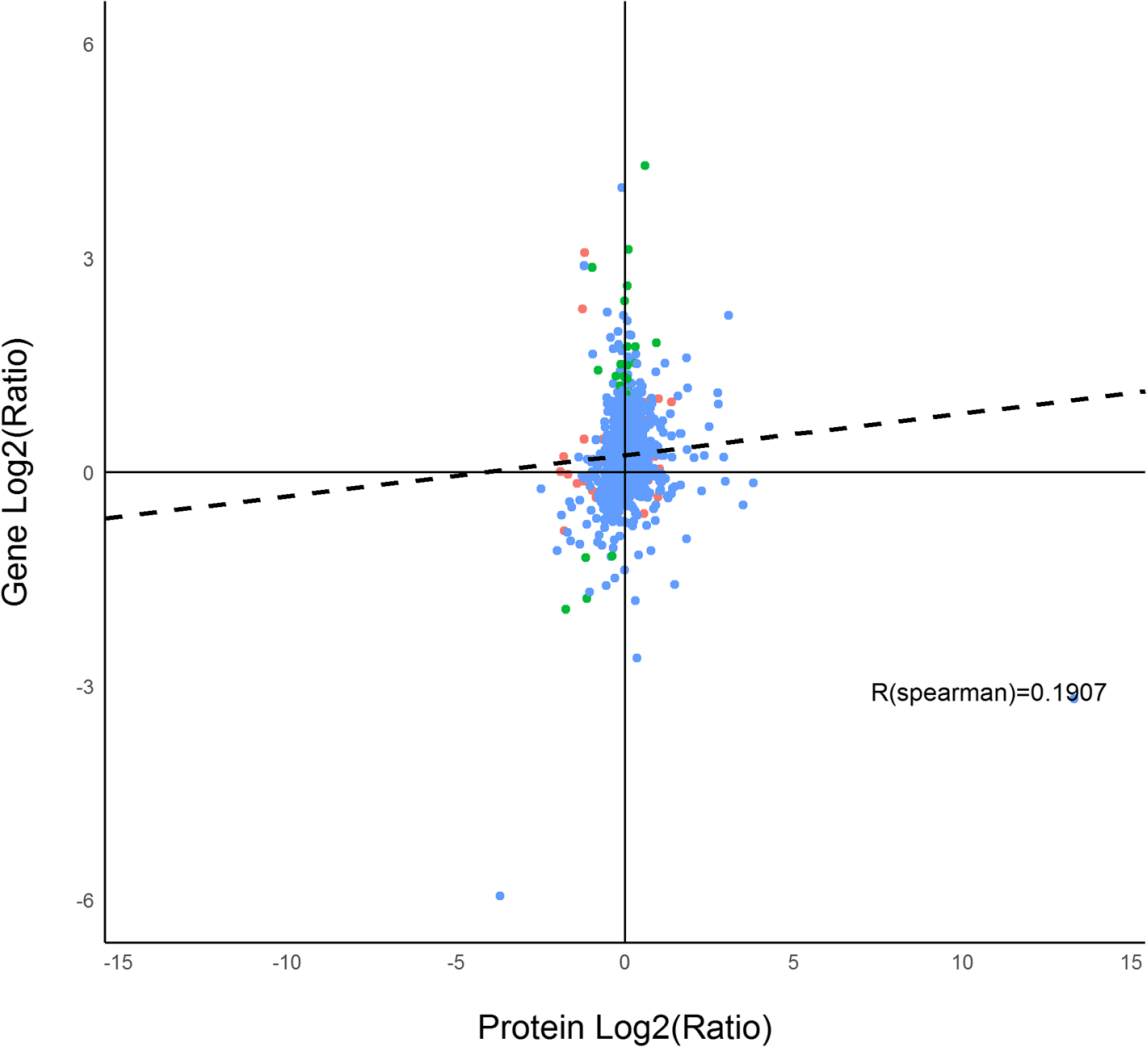
Association analysis of transcriptome and proteome differences between orange and white adductor muscle samples (protein fold changes > 1.2 and mRNA fold change > 2). Abscissa represents Log2-transformed protein fold changes; Ordinate represents Log2-mRNA fold changes. R (spearman) stands for spearman correlation coefficient. Red dots display significant changes of expression in protein levels only; green dots represent significant changes in mRNA expression levels only; blue dots denote no significant change of expression in either mRNA or proteins

The association analysis of the transcriptome and proteome data of white and orange adductor muscles revealed a nonlinear relationship between mRNA and protein expression with a Pearson’s correlation coefficient of 0.1907. Fifty-four genes were significantly expressed at protein expression level but not at mRNA level while 27 genes were significantly expressed at mRNA level but not at protein level. No genes were found to be statistically significantly expressed at both mRNA and protein levels (Fig. 6).

### RT PCR amplification and western blotting analysis of candidate genes and proteins related to pigments

The mRNA expression levels of 12 genes and proteins that may be directly or indirectly involved in coloration were evaluated by RT PCR in both orange and white adductor muscles (Fig. 4). The results showed that the expression levels of *Apolipoprotein, CYP450*, *tyrosinase*, and *VPS* were significantly higher in the orange adductor muscles than in the white adductor muscles (*P* < 0.05 or *P* < 0.01) while those of the rest of the genes (including *BCO1*, *BCO2*, *ApoA*, *ApoB*, *MTTP*, *SCAR*, *TIF*, and *RAB11A*) did not significantly differ between the orange and white adductor muscle samples (*P* > 0.05).

Western blotting analysis of *RAB11A* and *TIF* showed that the expression of these genes was significantly higher in the orange adductor muscles than in the white ones. Using a polyclonal antibody against *VPS*, a band (21 kDa) was only detected in orange adductor muscles, but not in the white ones (Fig. 5).

## Discussion

To explore the mechanisms of orange coloration in scallops, we compared the transcriptomes and proteomes of the orange adductor muscles of the QN Orange scallops and the white adductor muscles of the Bohai Red scallops. We then examined the differential expression of genes and proteins that may be involved in the accumulation of carotenoids and melanins, which are believed to be responsible for orange coloration in mollusks.

### DEGs and DEPs related to carotenoid accumulation

Previous study suggested that carotenoid accumulation may contribute to the orange coloration in scallops [14]. We also found that contents of carotenoids including pectenolone, pectenoxanthin and fucoxanthin in the QN Orange scallops are much higher than in the Bohai Red scallop (unpublished). Difference in carotenoid contents between orange and white muscle were thought to be caused by the differential expression of carotenoids absorbance or transport related genes in scallops [16, 17]. Therefore, in this study we first examined the potential mechanism of orange coloration caused by carotenoids.

A set of genes that may be involved in carotenoid metabolism, including BCO1, BCO2, ApoA, ApoB, MTTP and SCAR was identified in the orange adductor muscles of the QN Orange scallops. Among these genes, BCO1 and BCO2 are critical enzymes in deposition of carotenoids [18]. ApoA, ApoB and MTTP play a crucial role in transportation of carotenoids [16]. In pearl mussel *Hyriopsis cumingii*, the hcApo gene expression level existed positive correlation with the total carotenoid content [16]. SCAR recognizes lipoproteins binding carotenoids and facilitates the transfer of carotenoids [17]. In the noble scallop *Chlamys nobilis*, down-regulation of SCAR mRNA by RNA interference remarkably decreased blood carotenoid, providing compelling evidence that SCAR is an ideal candidate gene controlling carotenoid deposition and determining orange coloration [19]. However, mRNA expression levels of 6 genes (*BCO1, BCO2, ApoA, ApoB, MTTP* and *SCAR*) are not different between the white and orange adductor muscles as revealed by transcriptomic analyses and RT-PCR, suggesting that these genes are not the determining factors for the difference in carotenoids between the two types of adductor muscles.

It is interesting to find that 4 other genes, including *apolipophorin*, *TIF*, *VPS* and *CYP450*, which are outside of carotenoid pathway are found to express differentially between two types of adductor muscles in this study. These results suggest that carotenoids accumulation in orange adductor muscles may be controlled indirectly by those genes outside of carotenoid pathway in QN Orange scallops.

#### *Apolipophorin*

Like apolipoproteins, *Apolipophorin* is a member of the large lipid transfer protein superfamily that is responsible for trans-membrane transfer of lipids [20]. The expression of apolipoprotein has been reported to be related to carotenoid synthesis and shell coloration in pearl mussel [16]. Apolipophorins were light yellow in color presumably due to the presence of carotenoids [21]. As *apolipophorin* mRNA was expressed at higher levels in orange adductor muscle, it is possible that *apolipophorin* is also involved in carotenoid transport in the QN Orange scallop.

#### *TIF*

*TIF* encodes a translation initiation factor that functions in protein translation and has been found to increase β-carotene accumulation in scallops [22], although the underlying mechanism remains unclear. *TIF* may promote the translation of the enzymes of the carotenoid pathway. It is interesting to note that in this study, the elevation of *TIF* expression is only seen at the protein level but not at mRNA level. It is possible that only a portion of *TIF* transcripts were translated or maybe the protein level of *TIF* was affected by cotranslational, pretranslational, or posttranslational modifications.

#### *VPS*

*VPS* proteins are involved in the intracellular sorting and delivery of soluble vacuolar proteins. *VPS* has been reported to promote β-carotene deposition, as evidenced by the observation that *VPS* overexpression boosts the expression of genes related to the β-carotene pathway [22]. Furthermore, the gene homolog of *VPS* is necessary for the normal production of the pigment responsible for deep-orange eye coloration in *Drosophila* [23]. Proteome analysis detected the *VPS* protein only in orange adductor muscles but not in white adductor muscles. RT PCR analysis showed high levels of *VPS* mRNA in orange adductor muscle. This finding suggests that *VPS* exerts positive effects on carotenoid accumulation in adductor muscles.

#### *CYP450*

The high mRNA expression of *CYP450* in orange adductor muscles suggests that *CYP450* is also involved in the coloration in QN Orange scallops. In fact, *CYP450* has been reported to be responsible for the transformation of β-carotene through hydroxylation in *Haematococcus* [24]. In birds, carotenoid-related red-orange pigmentation in feathers is controlled by a *CYP450* gene cluster [25]. In marine snails, uroporphyrin is present in yellow-brown and pink-red foot tissues [26]. Uroporphyrin is a cyclic tetrapyrrole and can be either orange, yellow, red, blue, violet, green, or brown [8]. In the formation of uroporphyrin, *CYP450* catalyzes the oxidation of uroporphyrinogen to uroporphyrin [17]. Thus, *CYP450* also likely has an important role in the formation of orange coloration by interacting with several pigments.

### DEGs and DEPs related to melanin accumulation

Melanin may also contribute to the orange coloration in scallops. Presence of melanin results in yellow coloration in guppies (*Poecilia reticulata*) and yellow feathers in birds [10, 11]. In fact, melanin has been reported to contribute to shell pigmentation in mollusks [27, 28]. At the cellular level, melanocytes synthesize melanin within discrete organelles, termed melanosomes, which can be produced in varying sizes, densities, and numbers. The melanosomes can then be transported to other tissues. Quite a few melanogenic factors can modulate pigmentation in either a negative or positive fashion, which is quite complex at cellular level [29]. In this study, we identified three differentially expressed genes and proteins that may be involved in regulation of melanin accumulation in orange adductor muscles. These include *tyrosinase*, *RAB11A* and *CYP450*.

#### *Tyrosinase*

Although the complete pathways of melanin production and regulation are still not clear, numerous enzymes that are crucial for melanin synthesis and regulation in mollusks have been reported [8]. One of these enzymes is *tyrosinase. Tyrosinase* is upregulated in Pacific oysters with golden coloration [30]. The expression levels of *tyrosinase* in the mantles of the red-shelled Japanese scallop (*P. yessoensis*) are higher than those in the mantles of the white-shelled Japanese scallop [31]. Our transcriptome analysis revealed that *tyrosinase* is differentially expressed between orange and white adductor muscles. In addition, our RT PCR results illustrated that the mRNA abundance of *tyrosinase* is higher in orange adductor muscles than in white adductor muscles. These results suggest that *tyrosinase* may play an important role in coloration of QN Orange scallops, possibly through its enhancement on melanin synthesis.

#### *RAB11A*

Our data showed that *RAB11A* was expressed at high protein levels but not at mRNA level in orange adductor muscles. *RAB11A* belongs to the small Rab GTPase superfamily, which is associated with constitutive and regulated secretory pathways. It may be involved in protein transport and may play an important role in melanosome release and transport from melanocytes (www.uniprot.org/uniprot/P62491). Notably, several *RAB* genes can modulate pigmentation by controlling melanosome biogenesis in melanocytes [32]. The results in this study suggest *RAB11A* may be involved in adductor muscle coloration in QN Orange scallops possibly through posttranscriptional regulation.

#### *CYP450*

As discussed earlier, *CYP450* may affect orange coloration through the accumulation of different pigments in QN Orange scallops. In the Pacific oyster, *CYP450* has been found to be differentially expressed in oysters with black tissues, suggesting that the *CYP450* gene may be involved in melanogenesis [33]. Thus the possibility that *CYP450* affects orange coloration through the accumulation of melanin in QN Orange scallops can not be excluded.

### Transcriptome and proteome association analysis

In this study, no genes were found to be differentially expressed at both mRNA and protein levels. This phenomenon may be caused by the limitation in the current proteomic analyses which can reveal only limited number of DEPs. It is also possible that some genes are regulated at post-transcriptional level, not at mRNA level [34]. Some factors, such as microRNAs, may also regulate gene expression by binding to mRNA molecules and preventing translation, and eventually lead to decreased target protein expression [35].

## Conclusions

In conclusion, our transcriptomic and proteomic study revealed that multiple genes and proteins are differentially expressed between white and orange adductor muscles. Among these genes, *Apolipoprotein*, *VPO*, *TIF* and *CYP450* may affect β-carotene accumulation while other genes such as *tyrosinase*, *RAB11A* and *CYP450* may regulate the accumulation of melanin. Coloration in the orange adductor muscles may be controlled by the accumulation of carotenoids and melanin. These results provide new insights into the molecular mechanism underlying the adductor muscle coloration in the QN Orange scallop.

## Methods

### Tissues

QN Orange scallops with orange adductor muscles and Bohai Red scallops with white muscles were obtained from a scallop farm in Laizhou, Shandong Province. Adductor muscles of 9 animals of each strain were dissected and stored in liquid nitrogen individually until use. Three adductor muscles from each strain of scallops were pooled together for RNA extraction and proteins extraction, respectively. Three biological replicates for transcriptomic and proteomic analyses were prepared for total RNA and proteins.

### Transcriptome analysis

Total RNA was isolated with TRIzol reagent (Invitrogen, UK) in accordance with the manufacturer’s instructions. Then RNA purity and integrity were checked using NanoPhotometer® spectrophotometer (IMPLEN, CA, USA) and the RNA Nano 6000 Assay Kit of the Bioanalyzer 2100 system (Agilent Technologies, CA, USA), respectively. Subsequently, mRNA was purified with poly-T oligo-attached magnetic beads, fragmented by First Strand Synthesis Reaction Buffer (NEBNext, MA, USA), and reverse transcribed into cDNA. After purification, end repair and 3′-end adenylation, the cDNA fragments were ligated to NEBNext Adaptor with a hairpin loop structure. Final cDNA fragments with lengths of 250–300 bp were amplified by polymerase chain reactions (PCR) and the PCR products were purified on an AMPure XP system (Beckman Coulter, USA). The library quality was assessed using a Bioanalyzer 2100 system (Agilent, USA), and low-quality reads or reads containing poly-N or adapters were removed from the raw data to obtain high-quality clean reads. The bay scallop (*A. irradians irradians*) genome was used as reference genome (unpublished). Hisat2 v2.0.4 was employed for index calculation and to align the paired-end clean reads of the reference genome [36]. Gene expression level was quantified with HTSeq v0.9.1 [37] and FPKM [38].

DEGSeq R package 1.18.0 [39] was used to analyze differential gene expression in two groups with the filter criteria of false discovery rate < 0.05 and |log_2_FoldChange| > 1. Gene ontology (GO) terms and Kyoto Encyclopedia of Genes and Genomes database (KEGG) pathways were utilized to analyze differentially expressed genes (DEGs) with *P* < 0.05 [40].

### Label-free proteomic analysis

Proteins in the tissues were extracted with SDT lysis (4% sodium dodecyl sulfate, 100 mM Tris/Hcl, and 0.1 mM dichloro-diphenyl-tricgloroethane) and quantified by 12.5% sodium dodecyl sulfate-polyacrylamide gel electrophoresis. Protein enzymolysis was performed using a filter-aided sample preparation method [41]. Peptides were then desalted using C_18_ cartridges (66872-U Sigma), freeze-dehydrated, and redissolved in 40 μL of formic acid (0.1%). Each sample was separated by high-performance liquid chromatography with the liquid-phase system Easy nLC at the flow rate of 300 nL/min with nanoViper C18 column (Thermo Scientific Acclaim PepMap100, 100 μm × 2 cm, nanoViper C18) and C18-A2 column (Thermo Scientific EASY column, 10 cm, ID 75 μm, and 3 μm). Peptides were then analyzed using Q-Exactive (Thermo Scientific) with a survey scan ranging from 300 m/z to 1800 m/z. Full-scan mass spectra were obtained at an automatic gain control target of 1e6, a resolution of 70,000 at m/z 200, and a dynamic exclusion within 60s. After full scanning, high-energy collision dissociation fragmentation was captured with an underfill of 0.1% and a resolution of 17,500 at m/z 200, and normalized at a collision energy of 30 eV.

MaxQuant software 1.5.3.17 [42] was employed to analyze mass spectrum (MS) data. The parameters of the search engine were set as follows: Trypsin was selected as the digestive enzyme; the false discovery rate for protein and peptide was defined as < 1%; the fixed modification was carbamidomethyl; the main search was set at 6 ppm; and the MS tolerance search was set at 20 ppm.

GO terms were assigned using Basic Local Alignment Search Tool (BLAST). Differentially expressed proteins (DEPs) were mapped and annotated with Blast2GO. KEGG pathway analysis was employed to analyze the metabolic pathways present in the orange and white adductor muscles of scallops. DEPs were identified with a threshold of 0.05 and fold change of > 1.2.

### Association analyses of mRNA and protein expression levels

The relationships between protein and mRNA expression levels were assessed through Pearson correlation analysis. The graphical representations of scatter plots were constructed using R program.

### Quantitative real-time polymerase chain reaction analysis

Differentially expressed genes revealed by transcriptomic and proteomic analyses and genes that may be potentially related to adductor muscle coloration were evaluated by real-time polymerase chain reaction (RT PCR). These genes include *apolipophorin, CYP450*, *tyrosinase*, *RAB11A*, *TIF*, *VPS*, *BCO1*, *BCO2*, *MTTP*, *ApoA*, *ApoB*, and *SCAR*. Six orange and six white adductor muscles were selected for RT PCR analysis. Total RNA was isolated using TRIzol reagent (Invitrogen, UK). β-Actin was selected as the reference gene. The cDNA fragments were synthesized with Bestar qPCR RT Kit on a LightCycler 480 RT PCR instrument with Light-Cycler® 480 SYBR Green I Master Kit (Roche, Germany) according to the manufacturer’s instructions. The primers for RT PCR are listed in Table 2. The expression levels of genes were calculated using the comparative Ct method (ΔΔCt). The *t*-tests were performed using SPSS 17.0 for expression analysis and differences were considered significant if *P* < 0.05.

**Table 2:** Primer sequences for RT PCR

Gene name	Primer sequence (5′ to 3′)
*Apolipophorin*	F: CCACCTTTTGATGCTTTCGG
	R: GAGGACGATGGAGAAGTTACC
*CYP450*	F:GCCGCAATCATTCTGAAGTTG
	R: GAAATGCAGGTGTCAGCAAC
*VPS*	F: ATCCAGAGCAGAAGGTTGTAAC
	R: TTGTGAGTGTGACCAGAGATG
*TIF*	F: CCAGAAAGATGTAGGGTTCCG
	R: GTACCATCAAGTTCCTCTCCAG
*BCOD1*	F: GAGGGCGTAAAAGATGAGGTAC
	R: AGGTGCTTGAAGTTGTCCTG
*BCOD2*	F: AATGTTGCCGATCAGTACCC
	R: GCTTCATCGTTTTGGGATCG
*SCAR*	F: ATGGAGACCTGGCTATTTTGG
	R: CAGTCAATGCCATAAAACCCG
*MTTP*	F: CCAGAAAGATGTAGGGTTCCG
	R: GTACCATCAAGTTCCTCTCCAG
*ApoA*	F: ACACAGACCCAGGAAATGAAG
	R: TGTCCACATTCTCCTTGATCG
*ApoB*	F: CTTACCTGAACGTGACCTCG
	R: CTGAAAGTACATCTCCCTGCTC
*RAB11A*	F: TGGCTGGACGAGTTAAAAGAG
	R: TCAGGTCAGTTTTGTTCCCG
*Tyrosinase*	F: CCCTCCCAAGACATCAACAG
	R: CTCTGTAGATAGCACGCAGTTC
*β-Actin*	F: TATGCCCTCCCTCACGCTAT
	R: TTTCACTCTTTCCACCGGCTTT

### Western blotting analysis

Protein lysates isolated from orange adductor muscles and white adductor muscles were used for Western blot analysis as described by Funabara et al. [43]. Proteins were separated through SDS-PAGE and subsequently transferred to polyvinylidene fluoride membranes. The membranes were incubated overnight at 4 °C with primary antibodies. Tubulin was selected as the internal control. The primary antibodies were anti-tubulin (Abcam, ab6160, USA), anti-RAB11A (Abcam, ab65200, USA), anti-VPS (Abcam, ab98929, USA) and anti-TIF (abcam, ab230321, USA). Then, the membranes were rinsed thrice with TBS and were incubated with secondary antibodies (Beyotime, A0208) for 2 h at 37 °C. Gel documentation was observed using AlphaImager® HP (ProteinSimple, 92–13,824-00, USA). Band intensity was quantified with IPWIN software.

## Additional files

Additional file 1: **Table S1.** Summary of RNA-seq results. (DOCX 31 kb)

Additional file 2: **Table S2.** Basic characteristic of reads mapping to the reference genome. (DOCX 19 kb)

Additional file 3: **Table S3.** Differentially expressed proteins (DEPs) between the between orange (O) and white adductor muscle (W) samples by the lable free proteomics. (XLSX 22 kb)

## Abbreviations

*ApoA*: Apolipoprotein A
*ApoB*: Apolipoprotein B
*BCO1*: β-carotene15, 15′-monooxygenase
*BCO2*: β-carotene-9, 10′-oxygenase
*CYP450*: Cytochrome P450
*MTTP*: Microsomal triglyceride transfer protein
*RAB11A*: Ras-related protein Rab-11A
RT PCR: Real-time polymerase chain reaction
*SCAR*: Scavenger receptors
*TIF*: Translation initiation factor
*VPS*: Vacuolar protein sorting
