# Supplementary material for: Transcriptomic and proteomic analyses of genetic factors influencing adductor muscle coloration in QN Orange scallops
Source: BMC Genomics. 2019 May 9;20:363. doi: 10.1186/s12864-019-5717-y (PMC6509969; doi:10.1186/s12864-019-5717-y)
Supplement: Supplementary file 1 — Table S1. Summary of RNA-seq results. (DOCX 31 kb) [file 12864_2019_5717_MOESM1_ESM.docx]

Table S1 Summary of RNA-seq results

| Sample name | Raw reads | Clean reads | clean bases | Q20 (%) | Q30 (%) | GC content (%) |
| --- | --- | --- | --- | --- | --- | --- |
| W1 | 53387492 | 53040488 | 7.96G | 97.93 | 94.09 | 41.81 |
| W2 | 52598890 | 52257600 | 7.84G | 98.33 | 95.01 | 41.51 |
| W3 | 52616336 | 52248648 | 7.84G | 96.74 | 90.92 | 41.65 |
| O1 | 47591002 | 47288406 | 7.09G | 97.99 | 94.2 | 42.53 |
| O2 | 54794314 | 54435520 | 8.17G | 98.06 | 94.41 | 41.88 |
| O3 | 62428212 | 62076698 | 9.31G | 98.08 | 94.4 | 42.19 |
