# Supplementary material for: Transcriptomic and proteomic analyses of genetic factors influencing adductor muscle coloration in QN Orange scallops
Source: BMC Genomics. 2019 May 9;20:363. doi: 10.1186/s12864-019-5717-y (PMC6509969; doi:10.1186/s12864-019-5717-y)
Supplement: Supplementary file 2 — Table S2. Basic characteristic of reads mapping to the reference genome. (DOCX 19 kb) [file 12864_2019_5717_MOESM2_ESM.docx]

Table S2 Basic characteristic of reads mapping to the reference genome.

| Sample name | | Total reads | Total mapped | Exon | Intron | Intergenic |
| --- | --- | --- | --- | --- | --- | --- |
| W1 | 53040488 | | 48815410 (92.03%) | 74.00% | 5.40% | 20.60% |
| W2 | 52257600 | | 48408586 (92.63%) | 70.10% | 6.10% | 23.80% |
| W3 | 52248648 | | 47956046 (91.78%) | 72.20% | 5.20% | 22.60% |
| O1 | 47288406 | | 43860616 (92.75%) | 76.70% | 5.50% | 17.80% |
| O2 | 54435520 | | 50209559 (92.24%) | 74.20% | 6.00% | 19.80% |
| O3 | 62076698 | | 57579131 (92.75%) | 73.30% | 6.20% | 20.50% |
